# Mini review: Persister cell control strategies

**DOI:** 10.3389/fphar.2025.1706115

**Published:** 2025-10-15

**Authors:** Mohamad Javad Hashemi, Yousr Dhaouadi Khattab, Dacheng Ren

**Affiliations:** ^1^ Department of Biomedical and Chemical Engineering, Syracuse University, Syracuse, NY, United States; ^2^ Deparment of Biomedical Engineering, University of Tennessee, Knoxville, TN, United States

**Keywords:** persister bacteria, antibiotic, antibiotic persistence, combination therapy, AI-driven drug discovery

## Abstract

Bacterial persisters are growth-arrested cells with low metabolic activities, but have no genetic mutations compared to their parental cells. The dormant nature of persister cells enables them to tolerate high doses of conventional antibiotics and restart growth after the antibiotic is withdrawn, posing an important challenge to infection control. To promote more research in this important area, we present a concise review of current persister control strategies and discuss future opportunities.

## What are persister cells and why are they important?

Persister cells are growth-arrested phenotypic variants found in essentially all bacterial populations (Balaban et al., 2004; Lewis, 2010). Persisters can form both spontaneously (Balaban et al., 2019) and triggered by stressors such as pH change (Leimer et al., 2016), nutrient limitation (Nguyen et al., 2011), and antibiotic attack (Dewachter et al., 2019).

Conventional antibiotics were discovered based on bacterial growth inhibition. These molecules target and corrupt growth associated cellular processes, such as cell wall synthesis, DNA replication, and protein synthesis (Halawa et al., 2024). These processes require energy and are rather inactive in dormant persister cells (Germain et al., 2013; Amarh and Arthur, 2019); thus, conventional antibiotics commonly fail to eradicate persisters (Salcedo-Sora and Kell, 2020). Persister cells play an important role in recalcitrant diseases such as chronic lung infections of cystic fibrosis patients (Mulcahy et al., 2010), medical device-associated infections (Mishra et al., 2024), and Lyme disease (Sharma et al., 2015). Persisters also provide a reservoir of cells for the development of antibiotic-resistant strains over time (Santi et al., 2021). Thus, finding effective treatment for persister cells is a necessity for disease control.

In this mini-review, we briefly summarize the current strategies for persister control and discuss our view for future development. As a mini review, it is not a comprehensive overview with in-depth coverage of all related topics, but rather focuses on the principles and future perspectives. We are in debt to the scholars whose work is not cited here due to the limit of its scope.

## What strategies have been developed for killing persister cells?

**FIGURE 1:**
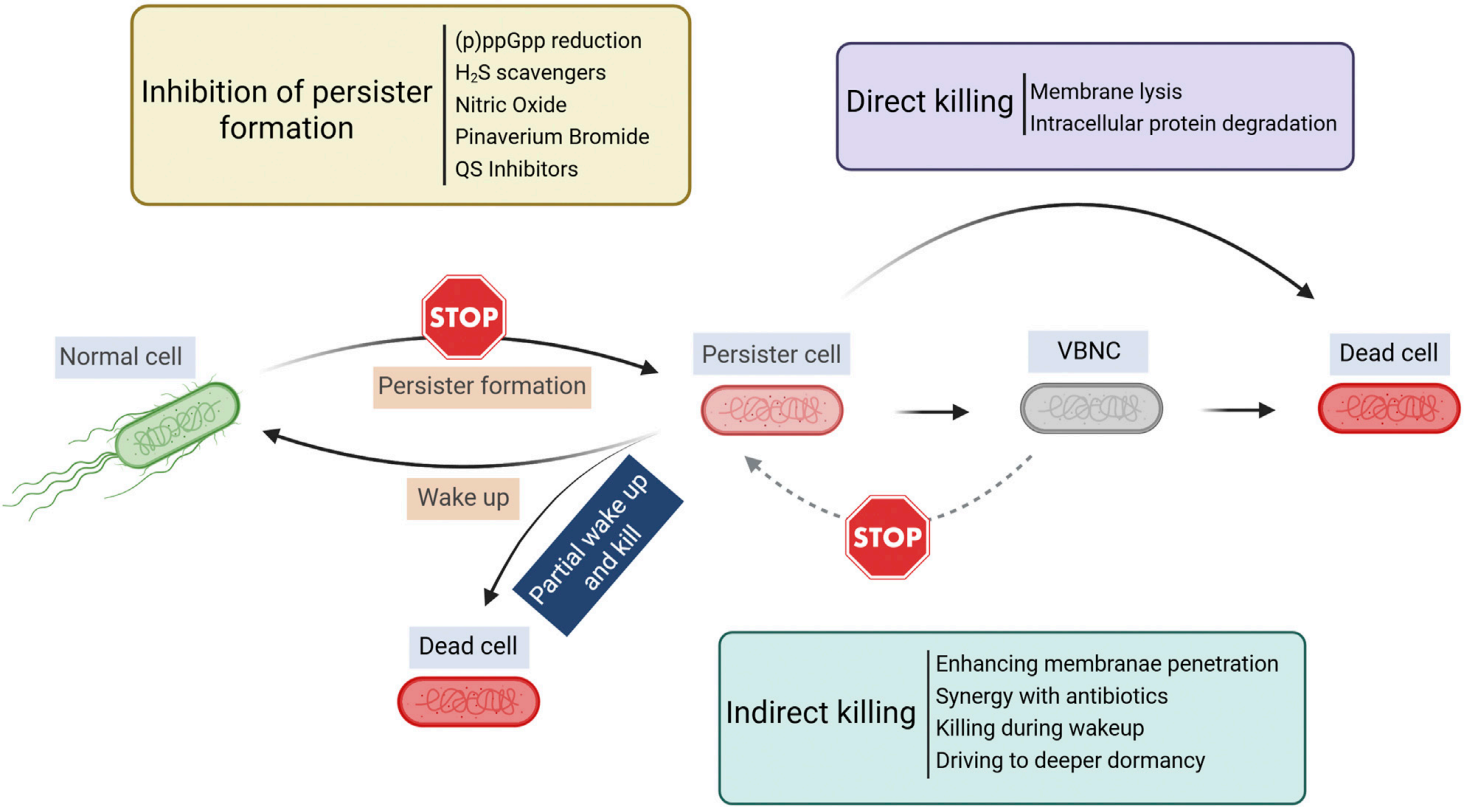
Overview of persister control strategies. Created in BioRender. Lab R. (2025) https://BioRender.com/krlwzbz.

Although persister cells are dormant and tolerant to most conventional antibiotics, persister cells still need to retain cell integrity and a capability to return to normal cells upon favorable changes in the environment. Thus, some targets of antimicrobials are retained in persister cells and new strategies can be developed leveraging unique characteristics of these metabolically dormant cells (Figure 1). The major strategies of persister control are summarized in Table 1 and discussed in the sections below.

**TABLE 1:** Major persister control strategies, advantages, and limitations (see specific sections above for references).

Strategy	Function	Advantages	Limitations
Direct killing of persister cells	Causes cell lysis by disrupting bacterial membranes or degrading proteins	Independent of bacterial growth state or metabolic activity	Off-target toxicity needs to be considered
Inhibiting persister formation	Alters bacterial metabolism, or inhibits QS	Bacteria-specific targets; reduces persister formation and antibiotic tolerance	May not be effective against already-formed persisters
Synergistic killing with antibiotics	Disrupts membrane integrity, enhances antibiotic uptake, alters metabolic state of persisters, or leverages other controls, e.g., electrochemical factors	Can eradicate both persister and actively growing bacterial cells	Needs to be effective across different bacterial species
Exploiting persister dormancy	Binds to intracellular targets to kill during wake-up or drives to deeper dormancy	Specifically target dormant cells	Limited research; mechanisms not fully understood

### Direct killing

#### Targeting cell membrane

Direct killing strategies attack growth-independent targets such as the cell membrane to cause cell lysis. Membrane damage can also generate lethal level of reactive oxygen species (ROS), contributing to persister killing (Gray et al., 2024). Multiple agents have shown activities against persister cells or dormant cells in general by directly damaging cell membranes. Some examples include 2D-24 (Bahar et al., 2015), AM-0016 (Mukherjee et al., 2016), XF-70 and XF-73 (Ooi et al., 2010; Board-Davies et al., 2023), SA-558 (Iu et al., 2023), thymol triphenylphosphine conjugates (TPP-Thy3) (Tang et al., 2024), and tea tree essential components (Nguyen et al., 2023). For example, SA-558 is a synthetic cation transporter. It disrupts bacterial homeostasis, leading to autolysis (Iu et al., 2023). Both XF-70 and XF-73 (Ooi et al., 2010; Board-Davies et al., 2023) are effective in killing non-dividing and slow-growing cells of *Staphylococcus aureus* by disrupting cell membranes. In addition, XF-73 generates ROS upon light activation, which oxidizes essential cellular components as a mechanism of its cidal effects (Maisch et al., 2005).

Additionally, synthesized cephalosporin derivatives (Chen et al., 2025) and red blood cell membrane-coated nanoparticles (Hb-Naf@RBCM NPs), which incorporate naftifine and oxygenated hemoglobin, effectively kill *S. aureus* persisters including those in biofilms (Zhu J. et al., 2024). Organo-soluble antimicrobial polymer nanocomposite (Patra et al., 2024) and semapimod (an anti-inflammatory drug) (Zheng et al., 2024) also exhibit anti-persister effects. Furthermore, cationic silver nanoparticle shelled nanodroplets (C-AgND) interact with the negatively charged components of the extracellular polymeric substance (EPS) layer, enabling effective killing of *S. aureus* persisters within biofilms (Bose and Das, 2025).

#### Other targets for direct killing

Pyrazinamide is a prodrug against *Mycobacterium tuberculosis* persisters. Its active form pyrazinoic acid disrupts membrane energetics, and binds to PanD (essential for coenzyme A biosynthesis) to trigger degradation of PanD by ClpC1-ClpP (Gopal et al., 2020; Conlon et al., 2013; Niu et al., 2024)Another example is ADEP4, a semi-synthetic acyldepsipeptide that binds to the ClpP protease and causes conformational changes, enabling ATP-independent protein degradation. This results in breakdown of over 400 intracellular proteins, including metabolic enzymes essential for persister wake-up. Their destruction renders the cells unable to recover and resume growth (Conlon et al., 2013; Niu et al., 2024).

Direct lysis of persisters is an effective approach as it does not require metabolic activities of the target cell. However, if an agent also affects mammalian membranes, it will limit its therapeutic potential due to off-target toxicity (Kaldalu et al., 2020). The field will benefit from more research on persister physiology and discovery of new persister-specific targets.

### Indirect killing

Most challenges posed by persister cells stem from their dormant nature. Conceptually, persisters can be eradicated either by preventing cells from entering dormancy or by inducing them to exit the persister state. Once reactivated, these cells become more susceptible to conventional antimicrobials. Alternatively, if a cell enters a deeper dormancy from which it cannot resuscitate, it effectively results in cell death. Exploiting shifts in dormancy depth may also create synergies with other treatments such as antibiotics.

#### Inhibit persister formation

Although the mechanism of persister formation is still not fully understood, multiple strategies have been shown to reduce persister formation (Balaban et al., 2019). For example, the pheromone cCf10 inhibits *Enterococcus faecalis* persister formation by reducing (p)ppGpp alarmone accumulation and maintaining its metabolically active state (Zhu L. et al., 2024). Another example is potentiation of persister killing using inhibitors of H_2_S biogenesis. $H_{2}S$ protects bacteria under stress conditions by scavenging free radicals and increasing the activity of antioxidant enzymes (Pal et al., 2018). Bacterial cystathionine g-lyase (bCSE) is the primary generator of H_2_S in *S*. *aureus* and *Pseudomonas aeruginosa*. CSE inhibitors were found to reduce biofilm formation and the number of persister cells, and potentiate antibiotics against both bacteria (Shatalin et al., 2021). Additionally, synthetic $H_{2}S$ scavengers were found to sensitize *S. aureus, P. aeruginosa, E. coli,* and MRSA persisters to gentamicin (Sun et al., 2024).

Also effective in preventing persister formation are nitric oxide (NO) that act as a metabolic disruptor (Orman and Brynildsen, 2016), and pinaverium bromide (PB) that disrupts PMF and generates ROS (Mao et al., 2023). In addition, some medium-chain saturated fatty acids have been shown to reduce persister formation, e.g., undecanoic acid, lauric acid, and N-tridecanoic acid (Jin et al., 2021).

While persister formation is controlled at the individual cell level, signaling between bacterial cells via quorum sensing (QS) has also been shown to affect persistence. QS is a bacterial cell-cell communication system that regulates multicellular behaviors in response to increase in cell density (Papenfort and Bassler, 2016). Möker et al. (2010) showed that the QS signals phenazine pyocyanin and the N-(3-oxododecanoyl)-L-homoserine lactone increase persister formation in *P.* aeruginosa by inducing oxidative stress and metabolic changes. Compounds that share a benzamide-benzimidazole backbone were found to bind to the QS regulator MvfR and inhibit MvfR regulon in *P. aeruginosa*, reducing its persister formation without affecting growth (Starkey et al., 2014; Maura and Rahme, 2017). Similarly, brominated furanones that are QS inhibitors reduce persister formation in *P. aeruginosa* (Pan et al., 2012).

### Synergy between antibiotics and other factors

Increasing membrane permeability has been shown to sensitize persister cells to antibiotics. For example, MB6-a potent methylazanediyl bisacetamide derivative-and two synthetic retinoids, CD437 and CD1530, bind to and embed in the MRSA lipid bilayer, thereby disrupting membrane integrity and increasing antibiotic uptake. Combined treatment of these compounds with gentamicin showed strong anti-persister activities (Kim et al., 2018; Heo et al., 2024). Similarly, Kim et al. (2019) reported MRSA persister cell killing by cotreatment with gentamicin and membrane active compounds bithionol and nTZDpa. Also functioning through membrane disruption are IMT-P8, a cell-penetrating peptide (Singh and Nandanwar, 2024), polymyxin B nonapeptide (PMBN) (Kim et al., 2024), and the polymyxin B derivative SPR741 (She et al., 2022). Moreover, Schmidt et al. (2014) engineered the aminoglycoside antibiotic tobramycin by adding 12 amino acids to convert it to the transporter sequence. The resultant molecule (Pentobra) exhibits strong activities in persister penetration and killing. In addition, gold nanocluster adjuvant, when combined with ofloxacin, could effectively kill persister cells (Cao et al., 2022). This was attributed to the ability of AuNC@CPP to hyperpolarize the cell membrane and disrupt the proton gradient (Cao et al., 2022). Dihydropyrrolidone-thiadiazole disrupts biofilm integrity and cell wall homeostasis by binding to cardiolipin, leading to cell wall disruption. Consistently, it showed synergistic effects with daptomycin in persister killing (Xiong et al., 2024).

Another strategy is to combine multiple antibiotics to eradicate persisters. Colistin paired with either aminoglycosides or ciprofloxacin is effective against persisters of *E. coli, K. pneumoniae, and A. baumannii*. Colistin is able to disrupt the outer membrane and then facilitate penetration of other antibiotics and increase their lethality (Bialvaei and Samadi Kafil, 2015; Chung and Ko, 2019).

Besides membrane disruption, antimicrobials can gain more penetration by manipulating membrane channels. An example is felodipine, an FDA approved dihydropyridine class of calcium channel blocker, which has low cytotoxicity to mammalian cells. When combined with gentamicin, the treatment dissipates MRSA membrane potential and increases cell membrane permeability. Additionally, felodipine reduces the TCA cycle and expression of aminoglycoside resistance proteins such as AacA-AphD. These led to killing MRSA persisters and biofilm cells in a mouse model (Zhang et al., 2022).

Disruption of proton motive force (PMF) also hinders efflux pump activities and increases the accumulation of certain antibiotics (Dewachter et al., 2019). One example is econazole, an FDA approved drug that dissipates the PMF and kills persister cells when used in combination with ampicillin, gentamycin, or ciprofloxacin. Cotreatment with econazole and ceftazidime was also found to kill tolerant bacterial populations *in vivo* (Wang et al., 2022). Additionally, exogenous adenosine and/or guanosine were found to increase accumulation of tetracycline in *Vibrio splendidus* persister cells, and cause cell death during the wake-up phase (Li et al., 2023).

Contrary to the approaches to reduce membrane integrity and PMF, increase in PMF and ATP could also reduce bacterial tolerance to antibiotics by increasing membrane energetics. Higher PMF, in addition to promoting the production of ROS, powers the uptake of antibiotics, especially the aminoglycosides, increasing their lethality (Lee et al., 2023). Compounds such as fumarate (Allison et al., 2011; Koeva et al., 2017), n-Butanol (Lv et al., 2022a), small molecule SA-558 (Iu et al., 2023), L-lysine (Deng et al., 2020) have anti-persister effects through increased antibiotic uptake. It is important to note that for the approaches that increase bacterial energetics, caution should be taken so that bacteria do not resume full growth and overpower the antimicrobials and the host immune system.

### Other mechanisms of synergy in persister killing

Besides chemical agents, hypoionic shock physically disrupts the cytoplasmic membrane, leading to activation of mechanosensitive channels. If effective antibiotics are applied during this process, it can result in substantial killing of persister cells (Lv et al., 2022b). In addition, low level electric currents have been found to increase persister killing by antibiotics. Electric currents can depolarize the cell membrane and facilitate passive diffusion of ions and antibiotics to persister cells (Niepa et al., 2012; 2016; 2017; Wang et al., 2020); Non-transducing phages have also been found to work in synergy with ciprofloxacin and ampicillin against cultures of uropathogenic *E. coli* (Vera-Mansilla et al., 2023).

### Leveraging the dormant nature of persister cells

Persisters are metabolically dormant and thus have reduced efflux activities. We reported recently that the agents capable of penetrating persister cells by passive diffusion can kill persister during wake up if the intracellular targets are available and if target binding is strong (Roy et al., 2021). These criteria can help guide the rational search for persister control agents. One example that fits these criteria is eravacycline, which is an amphiphilic antibiotic from the tetracycline family. It can enter persister cells through passive diffusion. Interestingly, it is more effective against persister cells than normal cells. This was attributed to the reduction of efflux in persister cells (Roy et al., 2021). Using eravacycline as a lead, we recently searched a small antimicrobial compound library with a chemoinformatic model. It is encouraging that 5 out of 11 candidate compounds identified through clustering are effective against persister cells (Roy et al., 2025). These findings provide helpful insights for finding new agents.

### Drive persister to deeper dormancy states

Previous research suggested that persisters and the viable but non-culturable (VBNC) state are not distinct, but rather stages along a dormancy spectrum (Ayrapetyan et al., 2018). A key driver of the transition from dormant persistence to difficult-to-resuscitate VBNC is protein aggregation during nutrient starvation (Dewachter et al., 2021). Thus, persister control may be achieved by driving cells to a deeper dormancy state like VBNC (Zhou et al., 2023). With more in-depth studies carried out in future, this could have major implications for antibiotic treatment strategies, chronic infection management and resuscitation protocols in the field. For example, it was found that lactate dehydrogenase (involved in pyruvate metabolism) promotes resuscitation of *E. coli* VBNCs, and cells with enhanced oxidative stress defense were more likely to resuscitate (Wagley et al., 2021). Finding new strategies/control agents that can stop VBNCs from resuscitation and/or going to deeper dormancy will also kill persisters. This is still a largely unexplored area.

## How to find better persister control agents?

Since persister cells are growth arrested, the search for persister killing agents should be focused on targets independent of metabolic activities. This requires new knowledge and strategies to identify these targets and new leads.

Artificial intelligence (AI) and machine learning (ML) are quickly transforming drug discovery and have been used in searching for better persister control agents (Wan et al., 2024; Zheng et al., 2024). AI, particularly deep learning models, enables researchers to efficiently screen millions of chemical compounds for antibacterial activities in a fraction of the time required by traditional methods. For example, large chemical libraries with more than 107 million molecules have been screened, leading to the discovery of new antibiotics including halicin (Stokes et al., 2020). In addition, deep learning-powered virtual screens were successfully applied to search for new agents against metabolically dormant bacteria, e.g., semapimod (Zheng et al., 2024). There is no doubt that the field will see more applications of AI models to accelerate both drug screening and the development of new models.

To better combat persistent infections, there are also needs for changes in drug discovery strategies. For persister control, this requires a shift from conventional MIC based screening to more specific targets in dormant cells. It is important to identify predictors for persister penetration, target binding, and killing activities based on new mechanisms. One method to gaining significant knowledge is the utilization of microfluidic platforms to isolate and manipulate individual bacterial cells in controlled environments (Luo et al., 2022). Integrating AI can automate imaging and predict cell fate, streamlining key steps in drug discovery.

To identify new targets in persister cells, we must obtain an in-depth understanding of persister formation mechanism and the true physiological stage of persister cells. There have been significant debates about the molecular mechanism of persister formation, which is partially attributed to the lack of robust methods to obtain persister cells in large quantities and the capability to separate the effects of persister formation itself from the effects of inducers applied to trigger persister formation. Heterogeneity in persister populations and the stochastic nature of formation is another challenge, which can possibly be solved by new persister isolation protocols and new technologies such as single cell RNAseq (Yan et al., 2024). To better eradicate persister cells, the drug of choice needs to bind the target strongly to overcome dormancy related slow killing kinetics. This can be achieved by modifying the drug molecule for stronger targeting including covalent binding. However, because these molecules are more active, the activity of the lead and possibility of undesired side effects must be carefully considered.

The field also need to address challenges in clinical translation of persister-targeting strategies. A central hurdle revolves around drug delivery of candidate compounds to niches where persisters reside, typically within biofilms (Wood et al., 2013), host tissues, or intracellularly within immune cells (Niu et al., 2024). Off-target toxicity is also a concern if the target is not bacteria specific and/or a high dosage of treatment agent is needed. Regulatory considerations may introduce further complexities such as fitting persister therapies within existing approved frameworks or introducing new regulations to fit the relapse and chronic nature of persistent infections (Defraine et al., 2018). Addressing these barriers is essential to moving persister therapies from the bench into clinical practice.

By exploiting the predictive power and speed of artificial intelligence and new biotech tools, scientists are now able to discover anti-persister drugs more efficiently. This marks a significant step forward in the global effort to develop the next-generation therapeutics for persistent infections.
